# Bedside personalized methods based on electrical impedance tomography or respiratory mechanics to set PEEP in ARDS and recruitment-to-inflation ratio: a physiologic study

**DOI:** 10.1186/s13613-023-01228-4

**Published:** 2024-01-05

**Authors:** Bertrand Pavlovsky, Christophe Desprez, Jean-Christophe Richard, Nicolas Fage, Arnaud Lesimple, Dara Chean, Antonin Courtais, Tommaso Mauri, Alain Mercat, François Beloncle

**Affiliations:** 1 Medical Intensive Care Unit, Vent’Lab, Angers University Hospital, University of Angers, 4 Rue Larrey, 49933 Angers Cedex 9, France; 2 Department of Anesthesia, Critical Care and Emergency, IRCCS (Institute for Treatment and Research, Ca’ Granda Maggiore Policlinico Hospital Foundation, Milan, Italy; 3 Department of Pathophysiology and Transplantation, University of Milan, Milan, Italy

**Keywords:** Recruitability, PEEP titration, Acute lung injury, Overdistension, Collapsus, Esophageal pressure

## Abstract

**Background:**

Various Positive End-Expiratory Pressure (PEEP) titration strategies have been proposed to optimize ventilation in patients with acute respiratory distress syndrome (ARDS). We aimed to compare PEEP titration strategies based on electrical impedance tomography (EIT) to methods derived from respiratory system mechanics with or without esophageal pressure measurements, in terms of PEEP levels and association with recruitability.

**Methods:**

Nineteen patients with ARDS were enrolled. Recruitability was assessed by the estimated Recruitment-to-Inflation ratio (R/I_est_) between PEEP 15 and 5 cmH_2_O. Then, a decremental PEEP trial from PEEP 20 to 5 cmH_2_O was performed. PEEP levels determined by the following strategies were studied: (1) plateau pressure 28–30 cmH_2_O (*Express*), (2) minimal positive expiratory transpulmonary pressure (*Positive P*_*L*_*e*), (3) center of ventilation closest to 0.5 (*CoV*) and (4) intersection of the EIT-based overdistension and lung collapse curves (*Crossing Point*). In addition, the PEEP levels determined by the *Crossing Point* strategy were assessed using different PEEP ranges during the decremental PEEP trial.

**Results:**

*Express* and *CoV* strategies led to higher PEEP levels than the *Positive P*_*L*_*e* and *Crossing Point* ones (17 [14–17], 20 [17–20], 8 [5–11], 10 [8–11] respectively, *p* < 0.001). For each strategy, there was no significant association between the optimal PEEP level and R/I_est_ (*Crossing Point*: *r*^2^ = 0.073, *p* = 0.263; *CoV*: *r*^2^ < 0.001, *p* = 0.941; *Express*: *r*^2^ < 0.001, *p* = 0.920; *Positive P*_*L*_*e*: *r*^2^ = 0.037, *p* = 0.461). The PEEP level obtained with the *Crossing Point* strategy was impacted by the PEEP range used during the decremental PEEP trial.

**Conclusions:**

*CoV* and *Express* strategies led to higher PEEP levels than the *Crossing Point* and *Positive P*_*L*_*e* strategies. Optimal PEEP levels proposed by these four methods were not associated with recruitability. Recruitability should be specifically assessed in ARDS patients to optimize PEEP titration.

**Supplementary Information:**

The online version contains supplementary material available at 10.1186/s13613-023-01228-4.

## Introduction

Acute respiratory distress syndrome (ARDS) is a major cause of mortality and morbidity in critically ill patients [1]. It is well established that a so-called *lung protective ventilation* strategy allows to improve patients’ outcomes [2]. This strategy is based on limited plateau pressure and tidal volume and adjusted positive end-expiratory pressure (PEEP) levels [2, 3]. There is, however, an important heterogeneity in terms of response to PEEP in patients with ARDS [4]. The concept of recruitability has been proposed to predict this response, in terms of “reopened” volume of flooded alveoli [4, 5].

Numerous PEEP titration strategies based on oxygenation, respiratory system mechanics or esophageal pressure measurements have been proposed, but none has been shown to be superior to any other [6–8]. These disappointing results might be explained by the inability of these PEEP titration strategies to deliver higher PEEP levels to patients with higher recruitability. Electrical impedance tomography (EIT) is an innovative technique using thoracic impedance to provide a real-time imaging of the distribution of gas in the lungs during ventilation [9, 10]. This allows the assessment of regional lung ventilation, including the re-opening of previously collapsed lung regions [11]. Different PEEP titration strategies have thus been proposed, based on the assessment with EIT of gas volume distribution between dependent and non-dependent regions of the lungs [12] and of the change in the amount of lung collapse and overdistension in response to an increase in PEEP [13]. The physiological effects of these EIT-based PEEP titration strategies are, however, poorly known. The interaction between these titration methods and recruitability has never been assessed.

This study hypothesis was that EIT-based strategies may lead to different PEEP levels from previously described bedside titration methods and allow to apply higher PEEP levels in patients with higher recruitability. In this exploratory, physiological study, we aimed to compare the PEEP levels determined by two EIT-based PEEP titration strategies and two respiratory mechanics-based methods and to assess the relation between these determined PEEP levels and the recruitability in patients with moderate to severe ARDS.

## Methods

### Study population

Nineteen adult patients, admitted to the Medical ICU of the University Hospital of Angers, France, from December 2019 to April 2020 were enrolled within 24 h after the diagnosis of ARDS defined according to the Berlin criteria. Ten patients with a COVID-19 associated ARDS (C-ARDS) have been included in a previously published study [14].

Ethics approval was obtained from the appropriate legal and ethical authorities (ethics committee of the University Hospital of Angers #2023-42). As the study reports data routinely acquired in usual care, signed informed consent was waived, according to local legislation.

### Patients’ installation and settings

Patients were deeply sedated by Midazolam and Fentanyl and paralyzed by continuous infusion of Cisatracurium. They were positioned in semi-recumbent position and ventilated in volume assist control mode using a Carescape R860 ventilator (General Electrics Healthcare ®, Madison, WI, USA). The following settings were applied to all patients: tidal volume 6 mL.kg^−1^ predicted body weight (PBW), respiratory rate set by the attending physician adjusted to maintain arterial pH above 7.30 (up to 35 min^−1^), FiO_2_ to obtain SpO_2_ > 94%.

EIT tracings were continuously recorded using a Pulmovista (Draeger ®, Lubeck, Germany) device. The tomography belt was positioned under armpits, between the third and the fifth intercostal space [9]. The anti-bedsore device mattress was turned off during the measurements to avoid interferences.

Esophageal pressure measurements were obtained with a specific nasogastric feeding tube equipped with an esophageal balloon (NutriVent®, Sidam, San Giacomo Roncole, Italy) connected to the ventilator (see the Additional file 1 for further information).

During all the study procedures, ventilator tracings including esophageal pressure measurements, EIT signals (ventilation distribution and lung volumes) were continuously recorded (further information on computed data are available in the Additional file 1: Table S1).

### Study protocol

Two distinct steps were consecutively conducted. The whole study protocol is summarized in the Additional file 1: Fig. S1.

#### Exploration of response to PEEP

PEEP level of 15, then 5 cm H_2_O was applied for 20 min. At the end of each period, inspiratory and expiratory pauses were performed, and arterial blood gases were obtained.

In addition, at PEEP 5 cmH_2_O, a low flow pressure volume curve was performed to detect a complete airway closure and measure airway opening pressure (AOP) [15].

#### Decremental PEEP trial

PEEP level was increased at 20 cmH_2_O then was progressively decreased by steps of 3 cmH_2_O every 3 min, until PEEP reached 5 cmH_2_O. Inspiratory and expiratory pauses were performed at the end of each step. The dynamic course of Center of Ventilation (CoV, the percentage of ventilation reaching the dorsal half of the lung), Pplat and expiratory transpulmonary pressure (P_L_e, difference between total PEEP and expiratory esophageal pressure) across the different PEEP levels were computed offline. EIT tracings allowed the estimation of lung ventilation distribution to assess the Center of Ventilation (CoV), and the reconstruction of Overdistension (OD) and Lung Collapse (LC) (see Additional file 1).

Based on this single trial, optimal PEEP levels for each PEEP strategy were defined as the one associated with: (1) Pplat between 28 and 30 cmH_2_O (*Express*) [6], (2) P_L_e between 0 and 2 cmH_2_O (*Positive P*_*L*_*e*) [16], (3) CoV closest to 50% (*CoV*) [12] and (4) intersection of LC and OD curves in a visual representation (*Crossing Point*) [13, 17, 18].

### Evaluation of recruitability

The recruitability was evaluated during the first phase with the estimated Recruitment-to-Inflation Ratio (R/I_est_), computed from an EIT-based measurement of the change in end-expiratory lung volume (ΔEELV_EIT_), as previously described [19].

ΔEELV_EIT_ was calculated by measuring the end-expiratory impedance gap between 15 and 5 cmH_2_O, corrected by the volume-impedance ratio [19]. In a sample of 9 patients from the present cohort, ΔEELV_EIT_ correlated well with ΔEELV measured by the single breath method (rho 0.716, *p* = 0.037, Additional file 1: Fig. S2).

The recruited volume (V_REC_) was computed as the difference between ΔEELV_EIT_ and the inflated volume related to the lung compliance at low PEEP, as follows: V_REC_ = ΔEELV_EIT_–(C_RS_-_PEEP5_ x ΔPEEP) [20]. ΔPEEP was the difference between the two PEEP levels (i.e., 15—5 = 10 cm H_2_O) or between the high PEEP level and the AOP in presence of complete airway closure at PEEP 5 cmH_2_O [5]. Recruited compliance (C_REC_) was computed as V_REC_/ΔPEEP. R/I_est_ was computed as the ratio between C_REC_ and C_RS_-_PEEP5_ [5].

To normalize the recruited volume on each patient weight, V_REC_/PBW was also calculated.

Finally, we measured the variation of lung collapse between PEEP 20 and 5 cmH2O (ΔCollapse_20-5_), as proposed by Jonkman et al. [18].

V_REC_, as V_REC_/PBW, could also be computed at each PEEP level from 5 cmH_2_O, by changing the ΔPEEP value by the following: PEEP_studied_ – 5 (or AOP if it reached a value above 5 cmH_2_O), in cmH_2_O.

### Evaluation of response to PEEP in terms of oxygenation and compliance

During the second study step, response on oxygenation (ΔPaO_2_/FiO_2_) was calculated as the difference between PaO_2_/FiO_2_ at PEEP 15 and 5 cmH_2_O, divided by the PaO_2_/FiO_2_ at 5 cmH_2_O. The same approach was used to assess the response on respiratory system compliance (ΔC_RS_).

### Statistical analysis

Data are expressed in number (percentage) or median [first-third quartile].

Patients were also pooled in groups according to: (1) measured R/I_est_ ratio, *higher R/I*_*est*_ and *lower R/I*_*est*_ groups were selected based on the median R/I_est_ value; (2) ΔPaO_2_/FiO2, also based on the cohort median value; (3) ΔC_RS_, also based on the cohort median value; (4) COVID associated ARDS vs. non-COVID ARDS.

Statistical comparisons were performed using a Mann–Whitney *U*-test for simple comparisons. For multiples comparisons, Friedman test or ANOVA were performed as appropriate; Bonferroni’s or Tukey’s correction were, respectively, applied to assess differences between two methods. Correlations between PEEP levels computed by the tested strategies and different recruitability and response to PEEP markers were performed using Spearman’s correlations.

All tests were performed with a type I error set at 0.05. The statistical analysis was performed using Prism (GraphPad Software v9.0, La Jolla, CA, USA).

## Results

### Patients’ characteristics

Baseline characteristics at inclusion of the 19 patients are summarized in the Table 1.

**Table 1:** Patients’ characteristics at baseline

	All patients *n* = 19
Demographics	
Age, years	64 [54–67]
Female gender (%)	4 (21)
BMI, kg.m^−2^	30.4 [24.8–33.6]
SAPS II score at ICU admission	45 [38–51]
ARDS etiology (%)	
*Pulmonary*	15 (79)
*Extra-pulmonary*	4 (21)
Mechanics and gas exchange at PEEP 5 cmH_2_O	
PaO_2_/FiO_2_, mmHg	120 [94–168]
PaCO_2_, mmHg	47 [39–52]
pH	7.33 [7.29–7.39]
Ventilatory Ratio	1.97 [1.64–2.45]
Tidal volume/PBW, mL.kg^−1^	6.1 [6.0–6.2]
Respiratory rate, min^−1^	30 [24–29]
PEEPtot, cmH_2_O	7.0 [5.5–8.0]
Plateau pressure, cmH_2_O	15 [14–18]
AOP > 5 cmH_2_O (%)	4 (21)
C_RS_, mL.cmH_2_O^−1^	50 [40–63]
C_CW_, mL.cmH_2_O^−1^	158 [131–219]
E_L_/E_RS_ ratio	0.70 [0.61–0.79]

*AOP* Airway Opening Pressure, *ARDS* Acute Respiratory Distress Syndrome, *BMI* Body Mass Index, *C*_*CW*_ chest wall compliance, *C*_*RS*_ respiratory system compliance, *E*_*L*_*/E*_*RS*_ lung elastance to respiratory system elastance ratio, *FiO*_*2*_ Fraction of inspired oxygen, *PaCO*_*2*_ Partial pressure of arterial carbon dioxide, *PaO*_*2*_ Partial pressure of arterial oxygen, *PEEPtot* total Positive End-Expiratory Pressure, *PLR* Potential for Lung Recruitment, *SAPS II* Simplified Acute Physiology Score II

According to the Berlin definition, severe, moderate, and mild ARDS were present at enrollment in 6 (32%), 10 (53%) and 3 (16%) patients, respectively.

Eleven patients (58%) died before day 28.

Esophageal pressure data were missing for one patient, due to a technical limitation.

### PEEP levels determined according to the different titration strategies

No adverse event was reported during the decremental PEEP trial.

**Fig. 1:**
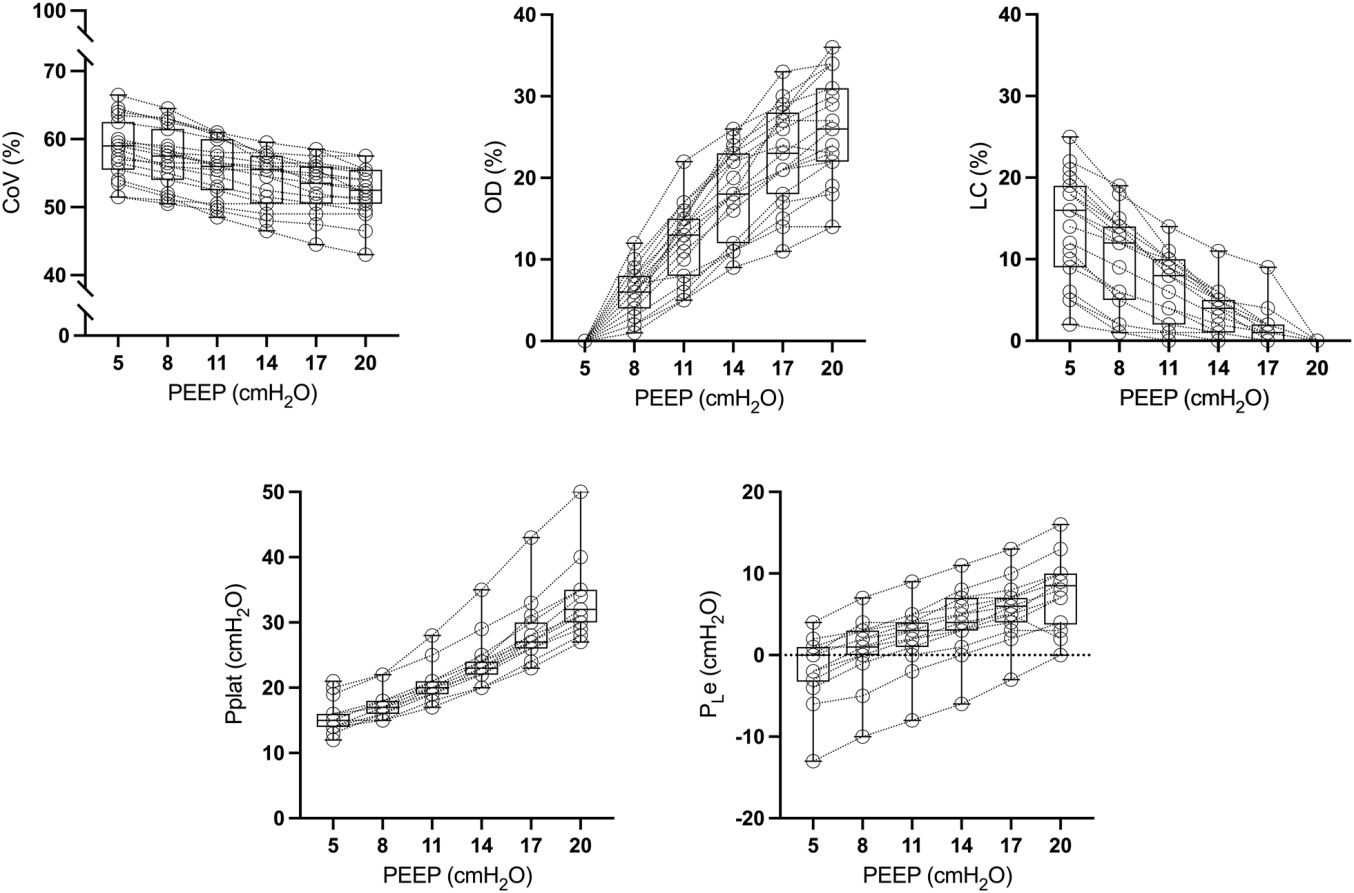
Changes in the studied physiologic variables during the decremental Positive End-Expiratory Pressure (PEEP) trial. **A** Center of Ventilation (CoV). **B** Overdistension (OD). **C** Lung Collapse (LC). **D** Plateau pressure (Pplat). **E** Tele-expiratory transpulmonary pressure (PLe). *Significantly different from PEEP 5 cmH_2_O (*p* < 0.05)

The different variables of interest assessed to define optimal PEEP levels according to the different titration strategies during the decremental PEEP trial are described in Fig. 1.

**Fig. 2:**
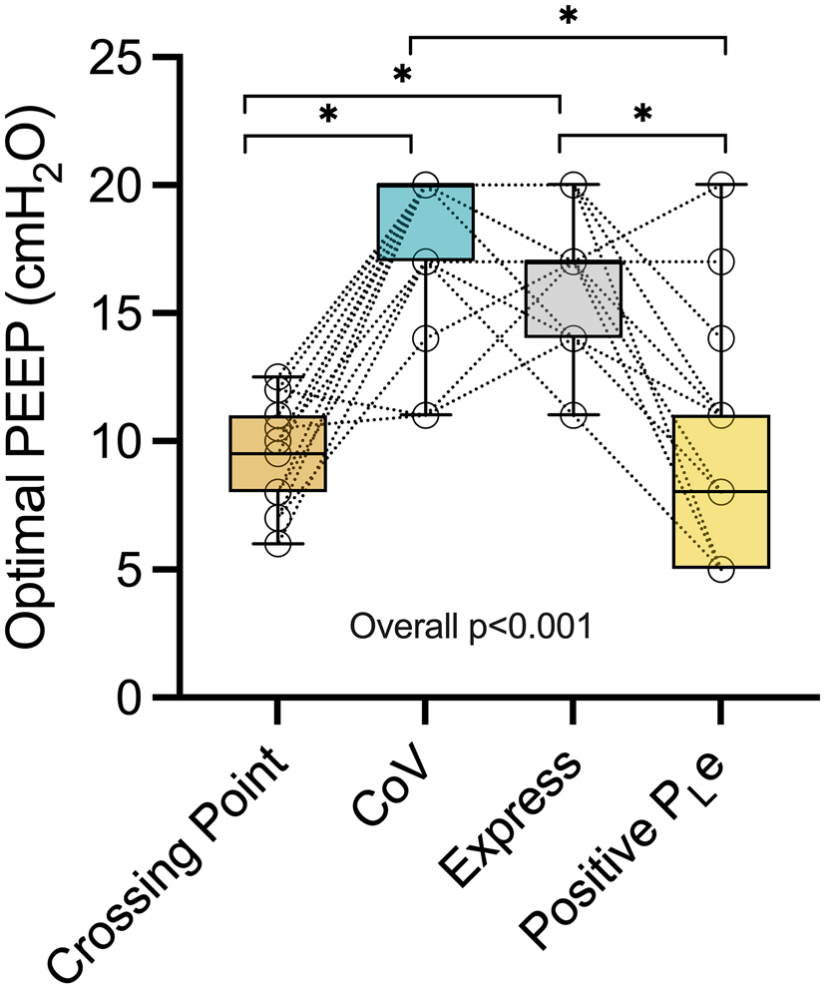
Positive End-Expiratory Pressure (PEEP) levels determined by the different titration strategies. *CoV* Center of Ventilation, *Crossing Point* Lung Collapse and Overdistension curves crossing point. *p < 0.05

In the whole cohort, the four PEEP titration strategies led to different PEEP levels (Fig. 2).

We observed no difference in the optimal PEEP levels between patients with C-ARDS and patients with ARDS of other etiologies in all the tested titration strategies (Additional file 1: Fig. S3).

### Optimal PEEP levels and recruitability

**Fig. 3:**
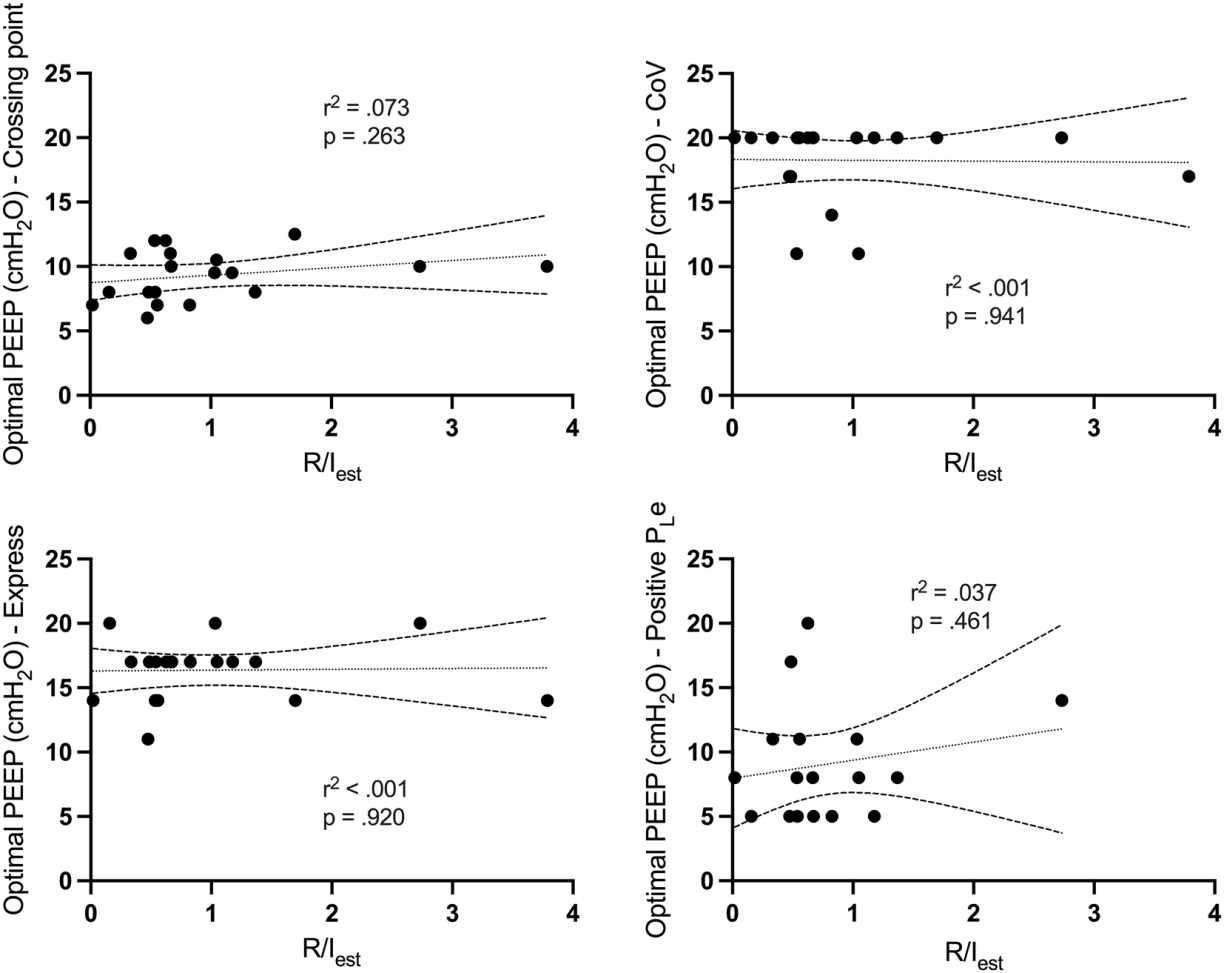
Correlations between Potential for Lung Recruitment assessed by recruitment-to-inflation ratio measured using electric impedance tomography (R/I_est_) and optimal Positive End-Expiratory Pressure levels (PEEP) levels determined by the different titration strategies**.** *CoV* Center of Ventilation, *Crossing Point* Lung Collapse and Overdistension curves crossing point

Patients in our cohort were characterized by a large variability of R/I_est_ (Fig. 3). Median R/I_est_ was 0.67 [0.48–1.18]. Median V_REC_/kg PBW was 4.9 [2.9–7.9] mL.kg^−1^ PBW.

There was no correlation between R/I_est_ and the optimal PEEP levels computed by the different methods (Fig. 3). Similar results were obtained with V_REC/_PBW (Additional file 1: Fig. S4). The comparison of ΔCollapse_20-5_ to the different optimal PEEP levels led to similar results, except for the *Crossing Point* method (Additional file 1: Fig. S5).

The determined PEEP levels did not differ between the *Higher* and *Lower* R/I_est_ ratio groups in all the tested PEEP titration strategies (Additional file 1: Fig. S6).

### Respiratory mechanics associated with each PEEP titration strategy

The different PEEP titration strategies led to differences in respiratory mechanics (Table 2). The *CoV* and *Express* strategies led to improved alveolar recruitment markers, with higher overdistention and lower compliance than the *Crossing Point* and *Positive P*_*L*_*e* methods.

**Table 2:** Respiratory mechanics and Electrical Impedance Tomography based measurements at the optimal Positive End-Expiratory Pressure (PEEP) levels computed for each tested PEEP titration strategy

	CoV	Crossing Point	Express	Positive P_L_e	ANOVA *p* value
PEEPtot, cmH_2_O	20 [19–20]*	11 [10–12]^†^	18 [16–19]*	7 [10–14]^†^	< .001
Pplat, cmH_2_O	31 [27–34]*	19 [17–20]^†^	27 [27–28]*	18 [14–21]^†^	< .001
P_L_e, cmH_2_O	7 [3–10]*	2 [0–3]^†^	6 [3–7]*	2 [1– 2]^†^	< .001
P_L_i, cmH_2_O	13 [10–22]*	7 [3–10]^†^	12 [10–15]*	7 [5–10]^†^	< .001
Pplat_L_, cmH_2_O	23 [16–27]*	14 [10–15]^†^	19 [16–22]*	11 [8–15]^†^	< .001
ΔP_RS_, cmH_2_O	11 [9–13]*	8 [7–10]^†^	9 [8–10]*	7 [7–10]^†^	< .001
C_RS_, mL. cmH_2_O^−1^	41 [28–50]*	54 [43–67]^†^	45 [42–56]*	59 [41–71]^†^	< .001
C_L_, mL. cmH_2_O^−1^	53 [34–67]*	75 [61–133]^†^	63 [53–88]*	77 [58–141]†	< .001
OD, %	24 [15–29]*	9 [6–13]^†^	21 [18–26]*	5 [0–14]†	< .001
LC, %	0 [0–1]*	9 [4–10]^†^	1 [0–2]*	9 [2–14]†	< .001
CoV, %	53 [51–56]*	57 [53–60]^†^	54 [51–57]*	56 [55–59]†	< .001
V_REC_ from PEEP 5 cmH_2_O, mL	511 [216–785]*	232 [140–401]^†^	517 [239–749]*	124 [0–155]^†^*	< .001
V_REC_/PBW from PEEP 5 cmH_2_O, mL.kg^−1^	7.4 [3.5–10.9]*	3.3 [2.3–4.9]^†^	7.5 [3.7–10.8]*	1.5 [0.0–2.6]^†^*	< .001

*C*_*L*_ Lung compliance, *CoV* Center of Ventilation, *C*_*RS*_ respiratory system compliance, *LC* Lung Collapse, *OD* OverDistension, *PEEPtot* total Positive End-Expiratory Pressure, *P*_*L*_*e* expiratory transpulmonary pressure, *P*_*L*_*i* inspiratory transpulmonary pressure, *Pplat* plateau pressure, *Pplat*_*L*_ plateau pressure applied to the lung, *ΔP*_*RS*_ respiratory system driving pressure. *Significantly different from Crossing Point method (*p* < 0.05), †Significantly different from CoV method (*p* < 0.05)

### Changes in oxygenation and respiratory system compliance in response to PEEP increase

Changes in oxygenation and C_RS_ after a PEEP increase from 5 to 15 cmH_2_O also covered a wide range: median ΔPaO_2_/FiO_2_ was 18.3 [− 3.8–37.1] % and median ΔC_RS_ − 5.6 [− 31.9–14.9] %.

Optimal PEEP levels defined by each strategy were not different between the patients for whom oxygenation significantly increased after an increase in PEEP and those for whom oxygenation did not increase (Additional file 1: Fig. S7A). Similar results were obtained by analyzing changes in C_RS_ (Additional file 1: Fig. S7B).

In addition, there was no statistical association between C_RS_ or PaO_2_/FiO_2_ at PEEP 5 cmH_2_O and R/I_est_ (*r*^2^ = 0.106, *p* = 0.174 and *r*^2^ = 0.165, *p* = 0.085, respectively) (Additional file 1: Fig. S8).

### Impact of the PEEP range of the decremental PEEP trial on Crossing Point computation

**Fig. 4:**
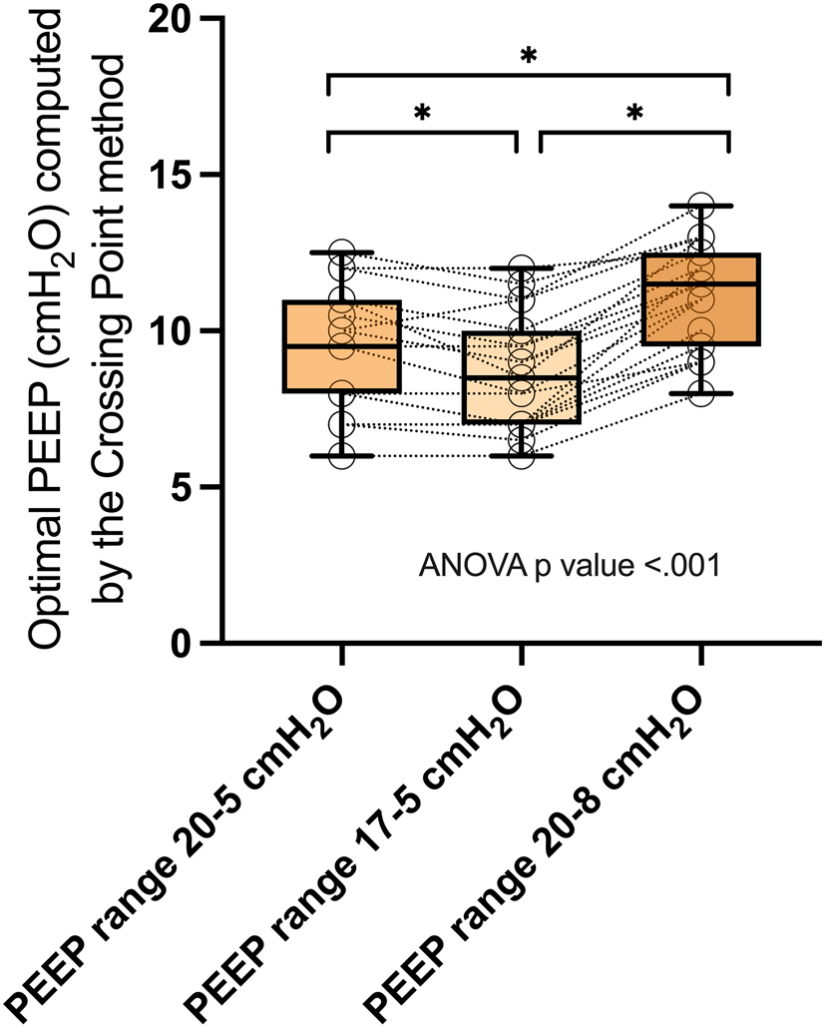
Optimal Positive End-Expiratory Pressure (PEEP) level according to the different PEEP ranges for *Crossing Point* method. *p < 0.01

Significant differences in PEEP levels determined according to the *Crossing Point* method were observed when the range of PEEP considered for the decremental PEEP trial and OD and LC curves reconstruction was modified (Fig. 4 and Additional file 1: Fig. S9).

## Discussion

The main findings of this study could be summarized as follows: (1) the four studied strategies lead to different optimal PEEP values, with *CoV* and *Express* strategies promoting similarly higher levels than the *Crossing Point* and *Positive P*_*L*_*e* ones. (2) There was no association between the PEEP levels obtained with these four different PEEP titration strategies and the recruitability assessed by R/I_est_. (3) Both *CoV* and *Express* associated PEEP levels are characterized by improved recruitment, but also increased overdistension and airway pressures; *Crossing Point* and *positive P*_*L*_*e* methods promote minimal overdistension and increased C_RS_ but have a lower impact on alveolar recruitment. (4) PEEP levels determined by the *Crossing Point* strategy depends on the PEEP range studied during the decremental PEEP trial.

### Association between lung recruitability and “optimal” PEEP levels

In the present series, optimal PEEP levels differed between the four tested strategies and were not related to R/I_est_ ratio. These results are consistent with other studies evaluating different EIT and esophageal pressure-based PEEP titration strategies [21–23]. In a physiological study using CT-scan to evaluate lung recruitability, neither the *Express* method nor the strategies based on esophageal pressure led to PEEP levels associated with recruitability [21]. Of note, patients with PaO_2_/FiO_2_ ratio between 200 and 300 mm Hg were included in this work. In another recent series in patients with C-ARDS, Perier et al. found no difference in PEEP levels determined by the *Crossing Point* method, in two subgroups defined according to lung recruitability assessed by the R/I ratio [22]. Similarly, Su et al. also reported an absence of correlation between the recruited volume and the optimal PEEP level measured by the *Crossing Point* method [23]. Interestingly, in another group of patients with severe ARDS, this strategy led to a better short-term mortality than a method based on the pressure–volume curve (PEEP set 2 cmH_2_O above the lower inflection point) [24]. However, in a recent large cohort characterized by a large heterogeneity among patients, there was an association between recruitability (measured by the ΔCollapse_20-5_ method using EIT), and the optimal PEEP level computed by the *Crossing Point* method, but also with ventilation homogeneity [18]. Importantly, in all these studies, the *Crossing Point* method allowed to set high PEEP levels, in populations characterized by a very high recruitability.

Elsewhere, in a population of post-surgical non-ARDS patients, the optimal PEEP level obtained with the CoV strategy was reached at the highest PEEP levels, suggesting a direct impact of PEEP on lung volume redistribution, even in patients without ARDS (i.e. not characterized by a high recruitability, albeit it was not assessed in this study) [25].

Importantly, to the best of our knowledge, our study is the first to compare respiratory mechanics, esophageal pressure and two distinct EIT-based titration strategies. The lack of association between lung recruitability and “optimal” PEEP levels observed in our study may contribute to explain the failure of large randomized controlled trials assessing PEEP titration strategies in ARDS [6–8].

### Towards a personalized PEEP titration strategy?

Among the four PEEP titration strategies tested in the present study, the rationale of *CoV* and *Express* strategies is mainly to target homogeneous ventilation and maximal recruitment, while *Crossing Point* and *positive P*_*L*_*e* strategies aim to combine “acceptable” recruitment and limited risk of overdistention during inspiration. Two recent studies compared the “silent spaces” strategy (aiming to reduce the total amount of unventilated lung volume) to strategies based on respiratory mechanics [26] or PEEP-FiO_2_ tables [27]. In these works, the “silent spaces” strategy was associated with higher PEEP levels, with improved recruitment, ventilation homogeneity and gas exchange.

Of note, the strategies based on lung homogeneity (*CoV*) and respiratory system mechanics (*Express*) led to higher PEEP levels than those based on the intersection of LC and OD curves and *positive P*_*L*_*e* strategies. Highly recruitable patients may benefit from strategies promoting recruitment, whereas minimal overdistension methods may be more appropriate for poorly recruitable patients. These results may thus be an incentive to specifically assess recruitability rather than systematically use any PEEP titration strategy. The choice of the optimal titration strategy may be discussed according to the most relevant awaited physiological benefit for the considered patient.

### Impact of PEEP range using the Crossing Point strategy

Our study shows that the PEEP level obtained with the *Crossing Point* strategy is impacted by the PEEP range used during the decremental PEEP trial. In patients with ARDS, four physiological studies aimed to study PEEP titration using *Crossing Point* method [17, 22, 28, 29]. In two series, optimal PEEP levels assessed by the *Crossing Point* method were higher than those determined by the same strategy in our study [17, 29]. This difference may be explained by the use of higher maximal PEEP levels during the decremental PEEP trial (from 40 to 5 cm H_2_O and from “at least” 24 to 10 cm H_2_O, respectively) [17, 29]. In the two studies using PEEP ranges close to the one used in our study (from 20 to 0 cm H_2_O, and from 6 to 18 cm H_2_O, respectively), optimal PEEP determined using the *Crossing Point* method were consistent with the levels observed in our cohort [22, 28].

These differences could be explained by the method of computation of lung collapse and overdistension, including the difference between the maximal compliance and the current compliance for each pixel at a given PEEP level, the wider the interval of PEEP levels studied, the higher the maximal compliance in some pixels [30]. This effect is illustrated in the Additional file 1: Fig. S8.

### Study limitations

There are some important limitations to this study: (1) the number of included patients is relatively small. (2) The study population is heterogeneous, with a large distribution of PaO_2_/FiO_2_ ratio at enrollment. In addition, more than half of the patients included in this study had a diagnosis of C-ARDS. No interaction between the ARDS etiology (i.e., COVID-19 vs. non-COVID-19) and determined PEEP levels was, however, observed. And differences in respiratory mechanics between C-ARDS and ARDS of other etiologies have been shown to be slight or non-existent and a large variety of phenotypes has been described in each group [31, 32]. (3) Respiratory system compliance was markedly high in our cohort, in comparison with other studies [1, 6, 7]. These values could be explained by the enrollment at the very early course of the disease [31]. (4) One could criticize the use of an EIT-based method to assess the R/I ratio. The V_REC_ computation based on EIT provides, however, results closely correlated to the inert gas dilution methods [33]. Moreover, the computation of R/I_est_ and the application of the *CoV* and *Crossing Point* strategies are based on different physiologic variables: R/I_est_ is based on ΔEELV, whereas *CoV* and *Crossing Point* strategies are based on tidal volume distribution. (5) Some physiologic effects may have not been perfectly controlled during the study. In particular, no recruitment maneuver with PEEP higher than 20 cmH_2_O was performed prior to the PEEP trial. And neither cardiac output nor mixed or central venous oxygen saturation was assessed in the study. However, no patient underwent any hemodynamic failure related to high PEEP levels during the PEEP trial. Finally, the short-term impact of the experiment on respiratory mechanics or gas exchange was not assessed.

## Conclusions

In this population of patients with ARDS, the *CoV* and *Express* strategies led to higher PEEP levels than the *Crossing Point* and *Positive P*_*L*_*e* strategy. Optimal PEEP levels proposed by these four methods were not associated with recruitability. Hence, recruitability should be specifically assessed in ARDS patients to optimize PEEP titration. The optimal method to set PEEP according to the R/I ratio remains to be determined.

### Supplementary Information

**Additional file 1:** **Table S1.** Presentation and computation of physiological parameters used in the study. **Figure S1.** Study protocol, represented by PEEP level across time.** Figure S2. **Correlation between estimated recruitment-to-inflation (R/I) ratio, based on change in end-expiratory lung volume measured by electrical impedance tomography (EIT) and R/I ratio based on the single breath trial (SBT) method.** Figure S3.** Optimal Positive End Expiratory Pressure (PEEP) computed according to the COVID-19 status. **Figure S4.** Correlations between recruitability assessed by recruited volume standardized for predicted body weight (VREC/PBW) and optimal PEEP levels. **Figure S5.** Correlations between recruitability assessed by the difference in lung collapse between PEEP 20 and 5 cmH2O (· Collapse20-5) and optimal PEEP levels. **Figure S6**. Optimal Positive End-Expiratory Pressure (PEEP) level computed after the decremental PEEP trial among patients with Lower (blue boxes, *n*=10) and Higher (red boxes, n=9) Recruitment-to-Inflation ratio estimated by Electrical Impedance Tomography (R/Iest). **Figure S7.** Optimal Positive End-Expiratory Pressure (PEEP) level computed after the decremental PEEP trial. **Figure S8.** Correlations between respiratory system compliance (CRS, panel **A**) or ratio between arterial pressure in dioxygen and inspired fraction in dioxygen (PaO2/FiO2, panel **B**) and recruitment-to-inflation ratio estimated by Electrical Impedance Tomography (R/Iest). **Figure S9. **Graphic representation of the mathematical variations in lung overdistension (OD) and collapsus (LC) induced by the changes in acquisition window.

## Data Availability

The datasets used and/or analyzed during the current study are available from the corresponding author on reasonable request.
