# Supplementary material for: Bedside personalized methods based on electrical impedance tomography or respiratory mechanics to set PEEP in ARDS and recruitment-to-inflation ratio: a physiologic study
Source: Ann Intensive Care. 2024 Jan 5;14:1. doi: 10.1186/s13613-023-01228-4 (PMC10769993; doi:10.1186/s13613-023-01228-4)

Bedside personalized methods based on electrical impedance tomography or respiratory mechanics to set PEEP in ARDS and recruitment-to-inflation ratio: a physiologic study

Bertrand Pavlovsky M.D., Christophe Desprez M.D., Jean-Christophe Richard M.D. Ph.D., Nicolas Fage M.D., Arnaud Lesimple M.S., Dara Chean M.D., Antonin Courtais M.D., Tommaso Mauri, M.D., Alain Mercat M.D. Ph.D., François Beloncle M.D. Ph.D

**Additional file 1**

# Methods

## Esophageal pressure

Esophageal pressure measurement was obtained with a specific nasogastric feeding tube equipped with esophageal balloon (NutriVent®, Sidam, San Giacomo Roncole, Italy) connected to the ventilator. The balloon was filled with air (between 2 and 4 mL).

The correct position of the esophageal balloon was checked by a chest x-ray. Once catheter was inserted, the tracing aspect was controlled, to avoid excessive cardiac artefacts [e1].

An occlusion test, as described by Baydur et al. [e1][e2] was provided. The operator performed a manual compression of the thorax during an expiratory or inspiratory occlusion of 5 seconds. The ratio between the ΔPes and the ΔPaw generated by the compression was calculated. The catheter positioning was validated with a ΔPes/ΔPaw ratio between 0.8 and 1.2.

An overview of the complete protocol is depicted in the eFigure 1.

## Precision on respiratory mechanics

Total PEEP (PEEPtot), Plateau Pressure (Pplat), expiratory and inspiratory esophageal pressures (Pes_e_ and Pes_i_, respectively) were measured offline on the ventilator derived airway and esophageal pressure tracings.

Transpulmonary inspiratory (P_L_i) and expiratory pressures (P_L_e), compliances and elastances of the respiratory system (C_RS_ and E_RS_), the chest wall (C_CW_ and E_CW_) and the lung (C_L_ and E_L_) were calculated.

The different computed physiological values are summarized in the eTable 1.

The R/I ratio following the single breath method was also computed in 8 patients [e3].

## Electrical impedance tomography

Center of Ventilation (CoV) was computed as the ratio between the tidal impedance variation in the dorsal lung regions (e.g., dependent zones) and the global tidal impedance variation [e4].

Overdistension (OD) and Lung Collapsus (LC) were computed using the following algorithms (equations (1) and (2), respectively) [e5].

1. OD(%) = $\sum_{i=1}^{n} \frac{{OD}_{(pixel)}i* {Cmax}_{(pixel)}i}{{Cmax}_{(pixel)}i}$ where OD_(pixel)_ = $\frac{C\max-Cz}{C\max}$

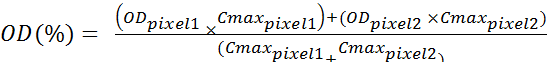


1. : LC(%) = $\sum_{i=1}^{n} \frac{{LC}_{(pixel)}i* {Cmax}_{(pixel)}i}{{Cmax}_{(pixel)}i}$ where LC_(pixel)_ = $\frac{C\max-Cz}{C\max}$

For each pixel, C_Z_ is the local compliance at the analyzed PEEP level, C_max_ the highest local compliance during the entire maneuver. Pixels are computed as overdistension at the PEEP levels higher than the level corresponding to C_max_, and conversely as lung collapse for the levels above [e6]. Pixel compliance was computed in VCV by using the measured driving pressure. The LC and OD curves were computed using three different offline analyses of the decremental PEEP trial (e.g. with PEEP ranges of 20-5 cmH_2_O, 17-5 cmH_2_O and 20-8 cmH_2_O), to assess the impact of the range variation on computed results.

## Data analysis

All the data from the ventilator (including airway pressure, esophageal pressure and flow time curves) were recorded using a computer connected to the ventilator and Ohmeda software (General Electrics Healthcare®); with a 25Hz sample rate. These data were then analyzed offline on Acqknowledge software (Biopac Systems ®, Goleta, CA, USA). The data from EIT device were analyzed off-line using Draeger EIT Data Analysis Tool V6.1 software (Draeger) and the non-commercially available MATLAB (Mathworks, Natick, USA) EITDiag software (Draeger).

# References

1. Akoumianaki E, Maggiore SM, Valenza F, Bellani G, Jubran A, Loring SH, Pelosi P, Talmor D, Grasso S, Chiumello D, Guérin C, Patroniti N, Ranieri VM, Gattinoni L, Nava S, Terragni PP, Pesenti A, Tobin M, Mancebo J, Brochard L; PLUG Working Group (Acute Respiratory Failure Section of the European Society of Intensive Care Medicine). The application of esophageal pressure measurement in patients with respiratory failure. Am J Respir Crit Care Med. 2014 Mar 1;189(5):520-31.
2. Baydur A, Behrakis PK, Zin WA, Jaeger M, Milic-Emili J. A simple method for assessing the validity of the esophageal balloon technique. Am Rev Respir Dis. 1982 Nov;126(5):788-91.
3. Chen L, Del Sorbo L, Grieco DL, Junhasavasdikul D, Rittayamai N, Soliman I, Sklar MC, Rauseo M, Ferguson ND, Fan E, Richard JM, Brochard L. Potential for Lung Recruitment Estimated by the Recruitment-to-Inflation Ratio in Acute Respiratory Distress Syndrome. A Clinical Trial. Am J Respir Crit Care Med. 2020 Jan 15;201(2):178-187.
4. Yoshida T, Piraino T, Lima CAS, Kavanagh BP, Amato MBP, Brochard L. Regional Ventilation Displayed by Electrical Impedance Tomography as an Incentive to Decrease PEEP. Am J Respir Crit Care Med. 2019 Jun 21.
5. Costa ELV, Borges JB, Melo A, Suarez-Sipmann F, Toufen C, Bohm SH, Amato MBP. Bedside estimation of recruitable alveolar collapse and hyperdistension by electrical impedance tomography. Intensive Care Med 2009 ; 35:1132–7.
6. Zhao Z, Lee LC, Chang MY, Frerichs I, Chang HT, Gow CH, Hsu YL, Möller K. The incidence and interpretation of large differences in EIT-based measures for PEEP titration in ARDS patients. J Clin Monit Comput. 2020 Oct;34(5):1005-1013.

# Additional file 1: Tables

| **Variable** | **Abbreviation** | **Formula** |
| --- | --- | --- |
| Respiratory system driving pressure | ΔP_RS_ | Pplat – PEEPtot* |
| Respiratory system compliance | C_RS_ | VTe / ΔP_RS_ |
| Respiratory system elastance | E_RS_ | ΔP_RS_ / VTe = 1 / C_RS_ |
| Transpulmonary inspiratory pressure | P_L_i | Pplat - Pes_i_ |
| Transpulmonary expiratory pressure | P_L_e | PEEPtot – Pes_e_ |
| Transpulmonary driving pressure | ΔP_L_ | P_L_i - P_L_e |
| Lung compliance | C_L_ | VTe / ΔP_L_ |
| Lung elastance | E_L_ | ΔP_L_ / VTe = 1 / C_L_ |
| Chest wall compliance | C_CW_ | 1 / E_CW_ |
| Lung elastance | E_CW_ | E_RS_ - E_L_ |
| Transpulmonary plateau pressure | Pplat_L_ | Pplat * E_L_/E_RS_ |

* Or Pplat – AOP if AOP>PEEPtot

**Table S1: Presentation and computation of physiological parameters used in the study.**

# Additional file 1: Figures

**Figure S1. Study protocol, represented by PEEP level across time.** ABG: arterial blood gas, PEEP: Positive End Expiratory Pressure.

**Figure S2. Correlation between estimated recruitment-to-inflation (R/I) ratio, based on change in end-expiratory lung volume measured by electrical impedance tomography (EIT) and R/I ratio based on the single breath trial (SBT) method.** Both methods were available only for COVID-19 patients (n=10), rho=0.716, p=0.037.

**Figure S3. Optimal Positive End Expiratory Pressure (PEEP) computed according to the COVID-19 status.** COVID-19 n=10, non-COVID-19 n=9.

**Figure S4. Correlations between recruitability assessed by recruited volume standardized for predicted body weight (V_REC_/PBW) and optimal PEEP levels.** *CoV: Center of Ventilation, P_L_e: transpulmonary tele-expiratory pressure.*

**Figure S5. Correlations between recruitability assessed by the difference in lung collapse between PEEP 20 and 5 cmH_2_O (ΔCollapse_20-5_) and optimal PEEP levels.** *CoV: Center of Ventilation, P_L_e: transpulmonary tele-expiratory pressure.*

**Figure S6.** **Optimal Positive End-Expiratory Pressure (PEEP) level computed after the decremental PEEP trial** among patients with Lower (blue boxes, n=10) and Higher (red boxes, n=9) Recruitment-to-Inflation ratio estimated by Electrical Impedance Tomography (R/I_est_). *CoV: center of ventilation, P_L_e: expiratory transpulmonary pressure.*

**Figure S7.** **Optimal Positive End-Expiratory Pressure (PEEP) level computed after the decremental PEEP trial**. A. Among patients “non-responders” to PEEP change in terms of oxygenation (cyan boxes, n=9) and “responders” to PEEP (orange boxes, n=10), defined by the ΔPaO_2_/FiO_2_ (≤ or > +20%), between PEEP 15 and 5 cmH_2_O. B. Among patients “non-responders” to PEEP change in terms of compliance (green boxes, n=10) and “responders” to PEEP (yellow boxes, n=9), defined by the ΔC_RS_ (≤ or > 0%), between PEEP 15 and 5 cmH_2_O. *CoV: center of ventilation, P_L_e: expiratory transpulmonary pressure.*

**Figure S8. Correlations between respiratory system compliance (C_RS_, panel A) or ratio between arterial pressure in dioxygen and inspired fraction in dioxygen (PaO_2_/FiO_2_, panel B) and recruitment-to-inflation ratio estimated by Electrical Impedance Tomography (R/I_est_).**

**Figure S9.** **Graphic representation of the mathematical variations in lung overdistension (OD) and collapsus (LC) induced by the changes in acquisition window.** P1 profile represents all the pixels with an increase in local compliance when PEEP level falls, and P2 profile the pixels showing a decrease in local compliance at the same time. At a given PEEP level, here PEEPset, each pixel have a local compliance (Cz), and a maximal compliance during the decremental PEEP trial (Cmax). When a full window acquisition is performed (acquisition 1), the pixel 2 has a higher Cmax (Cmax_p2(Acq1)_) than in a partial window acquisition (acquisition 2 ; Cmax_p2(Acq2)_), here with a suppression of the highest PEEP levels (blue area).

**Figure S1**


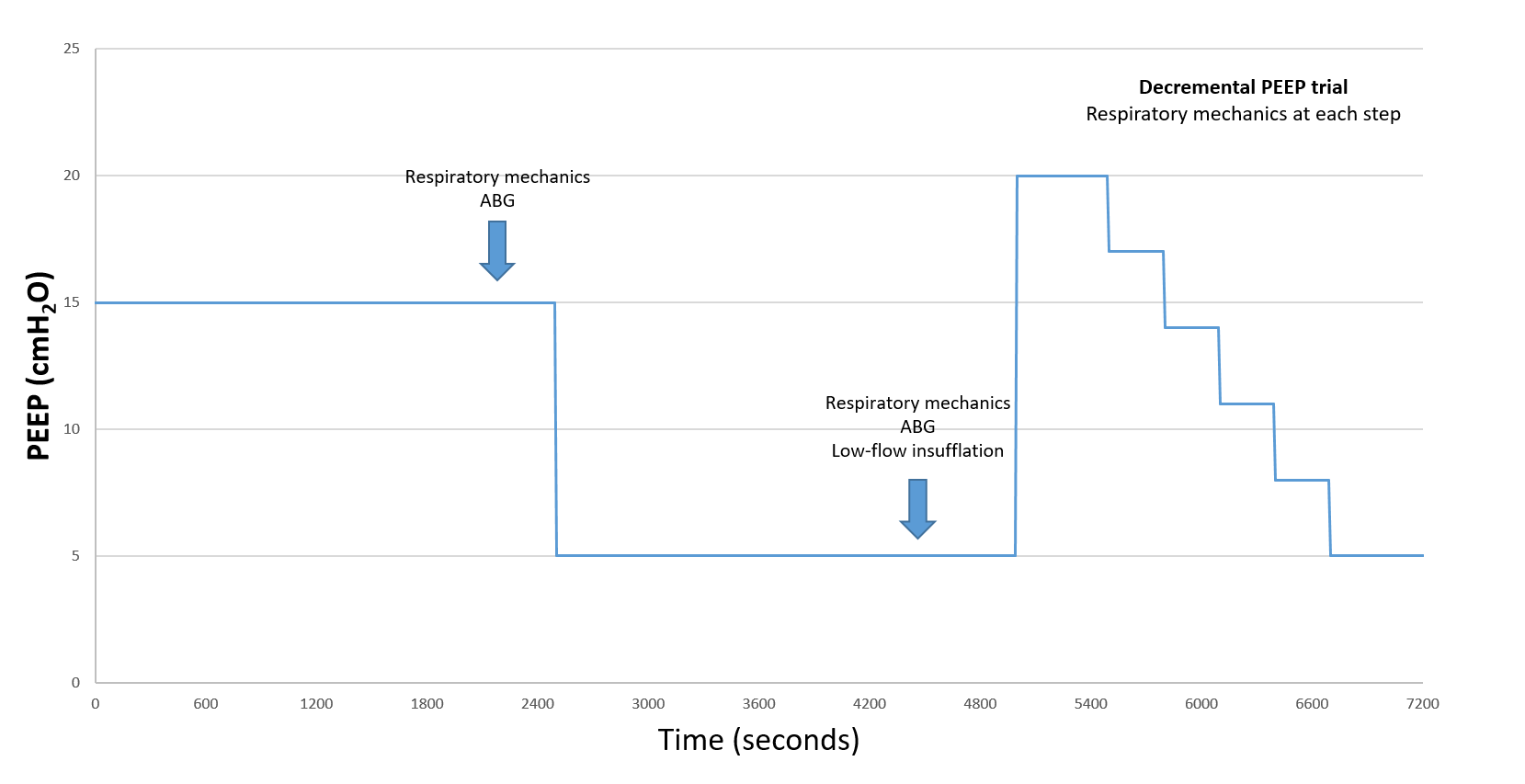


**Figure S2**

**
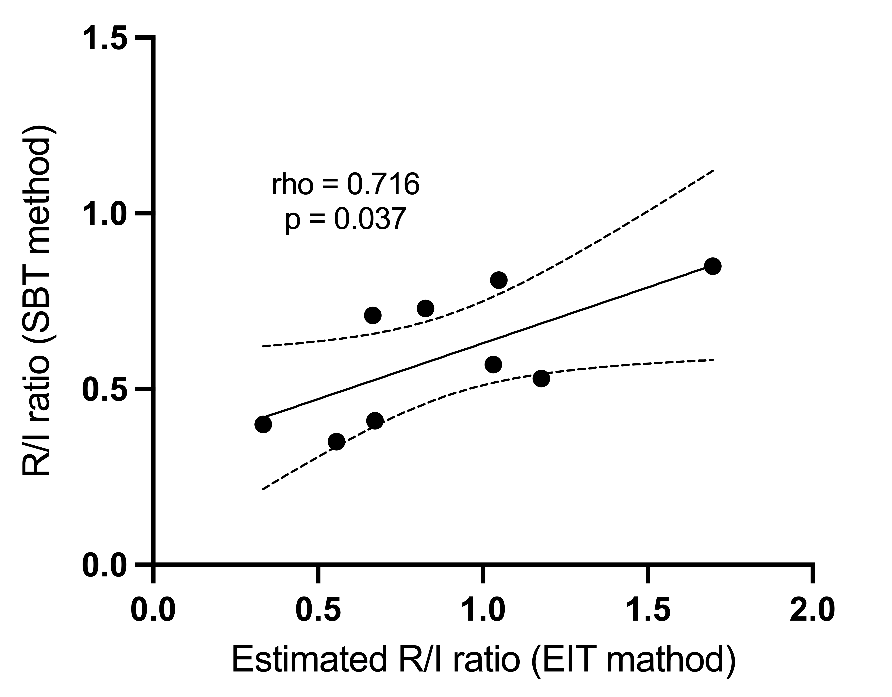
**

**Figure S3**


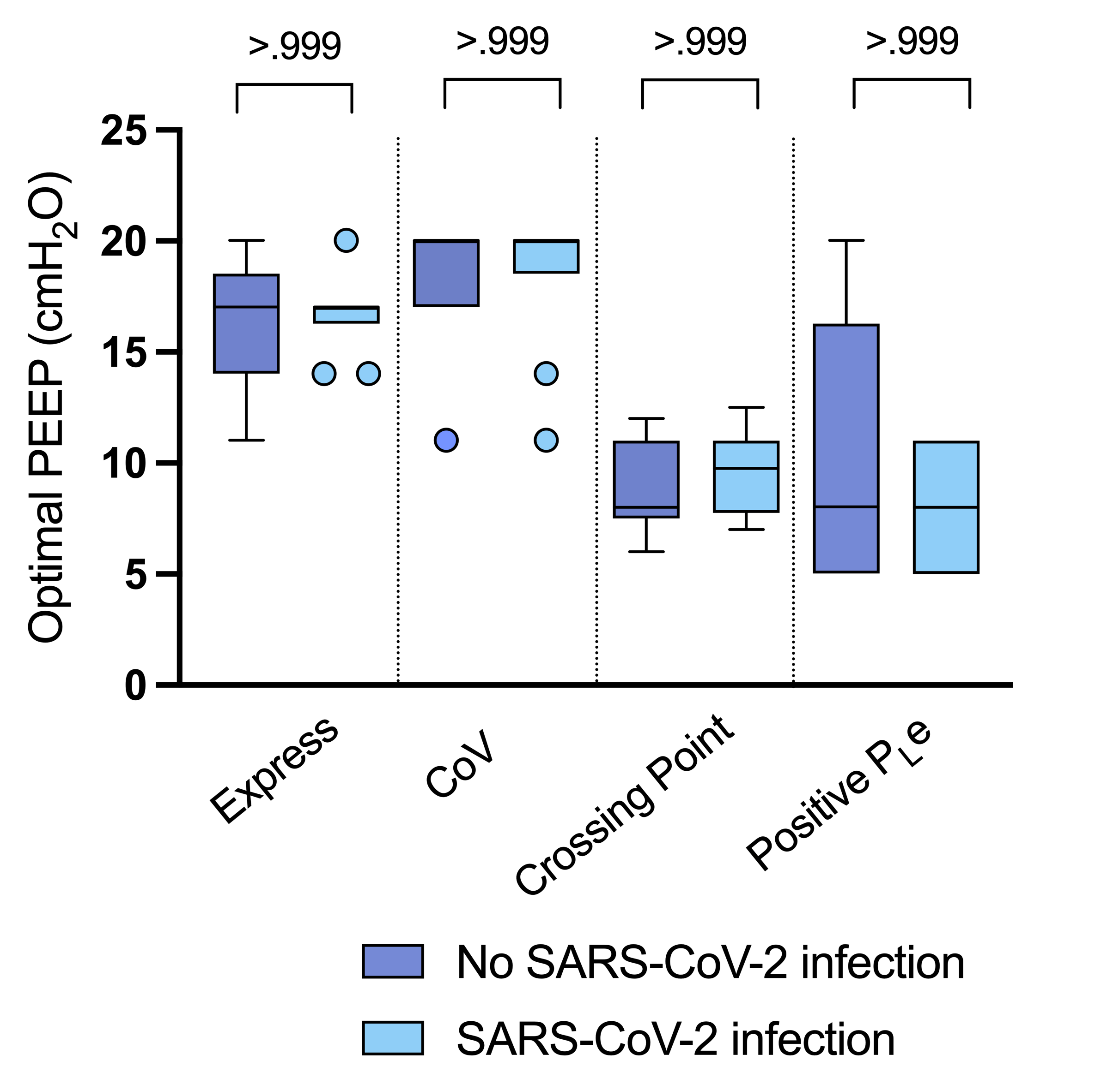


**Figure S4**

**
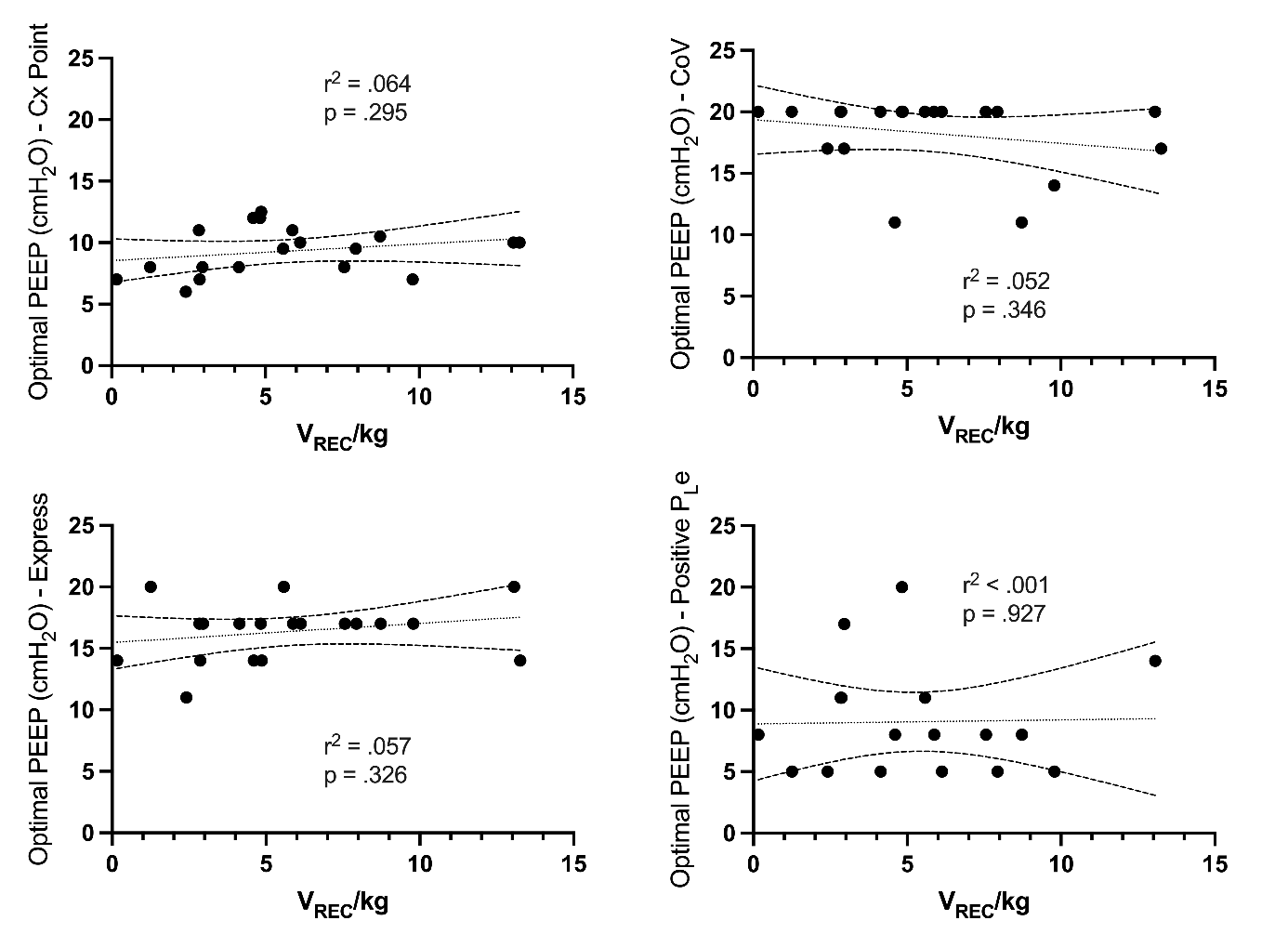
**

**Figure S5**

**
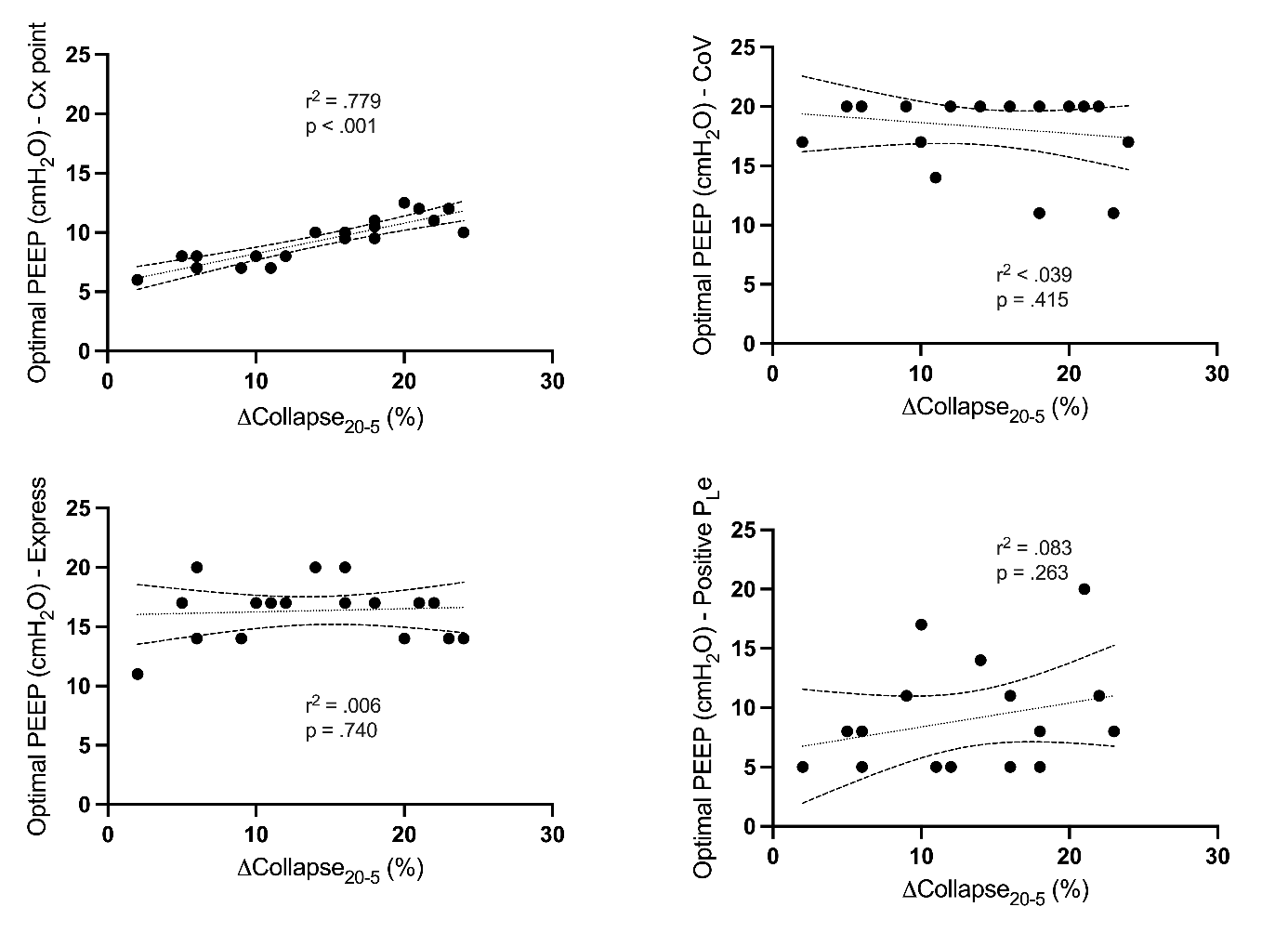
**

**Figure S6**


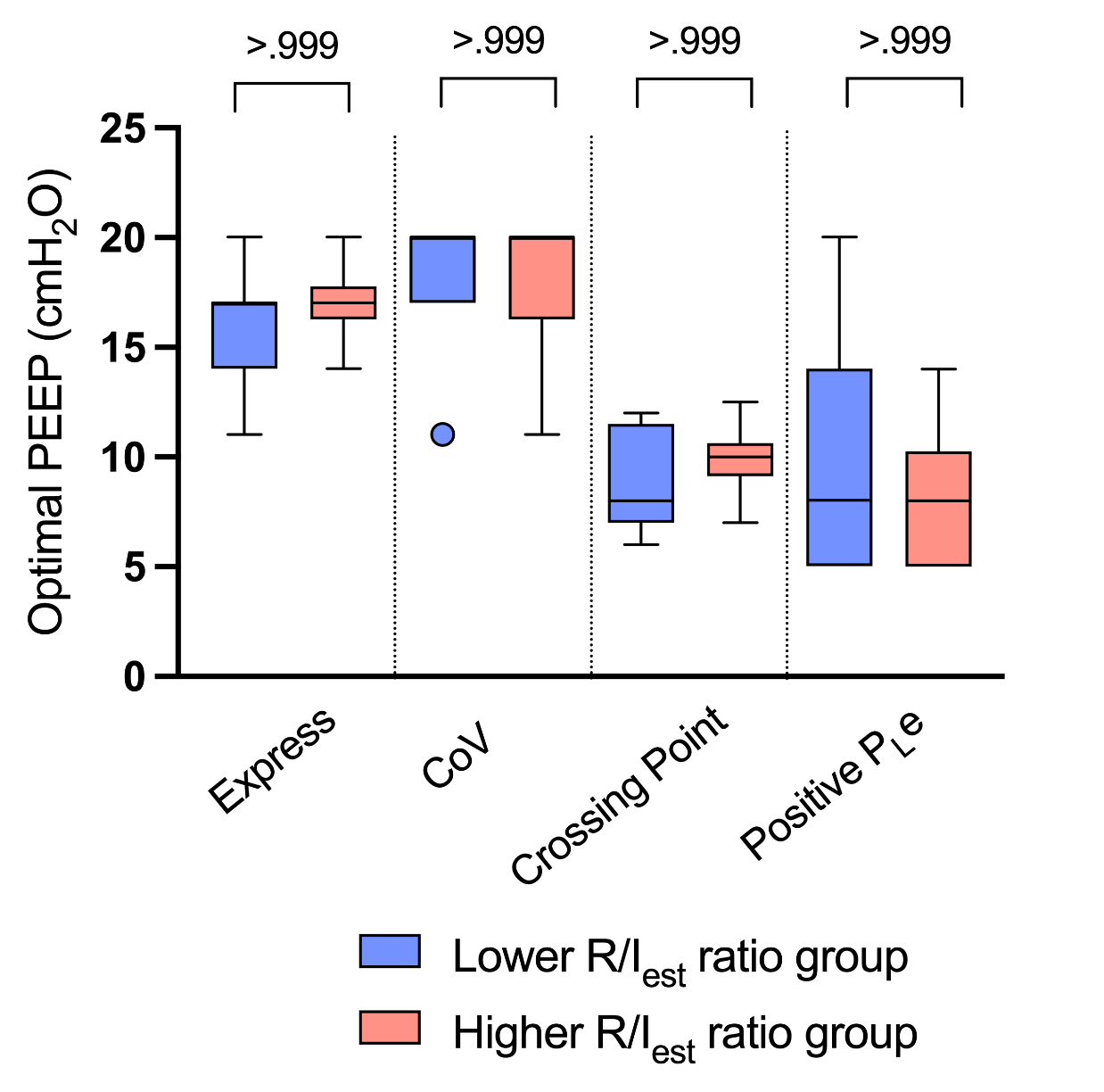


**Figure S7**

**
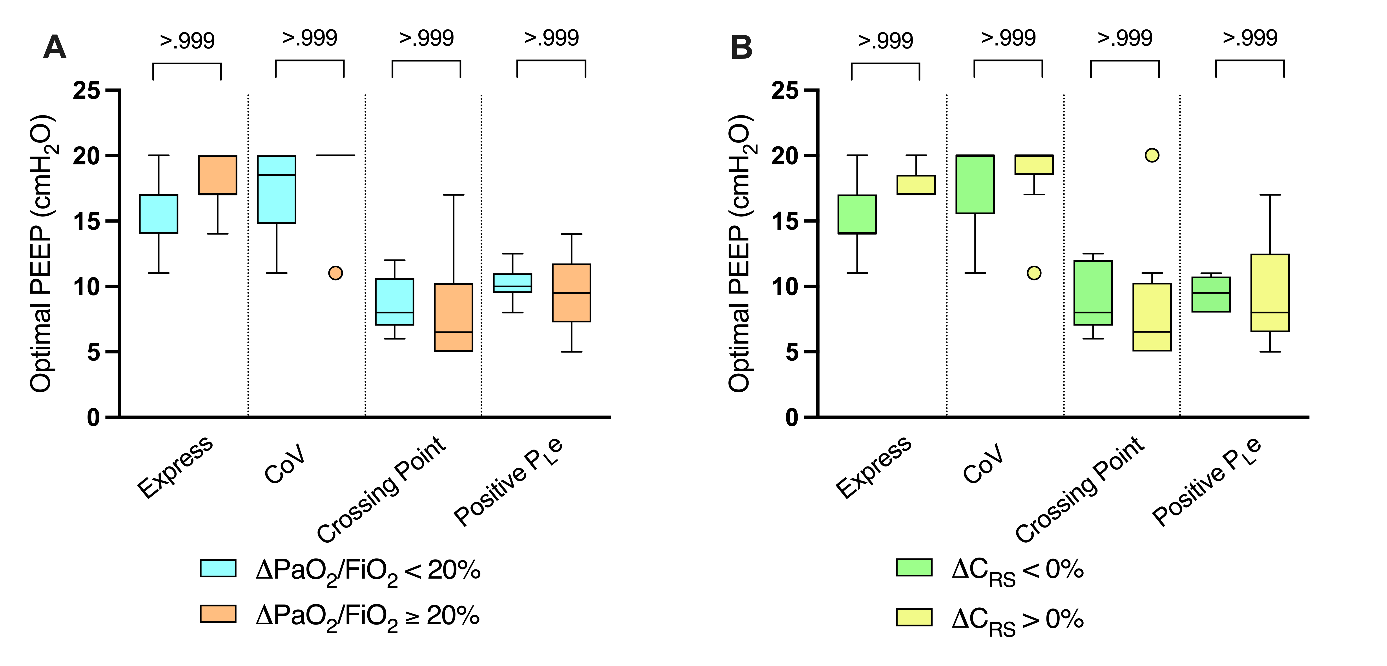
**

**Figure S8**

**
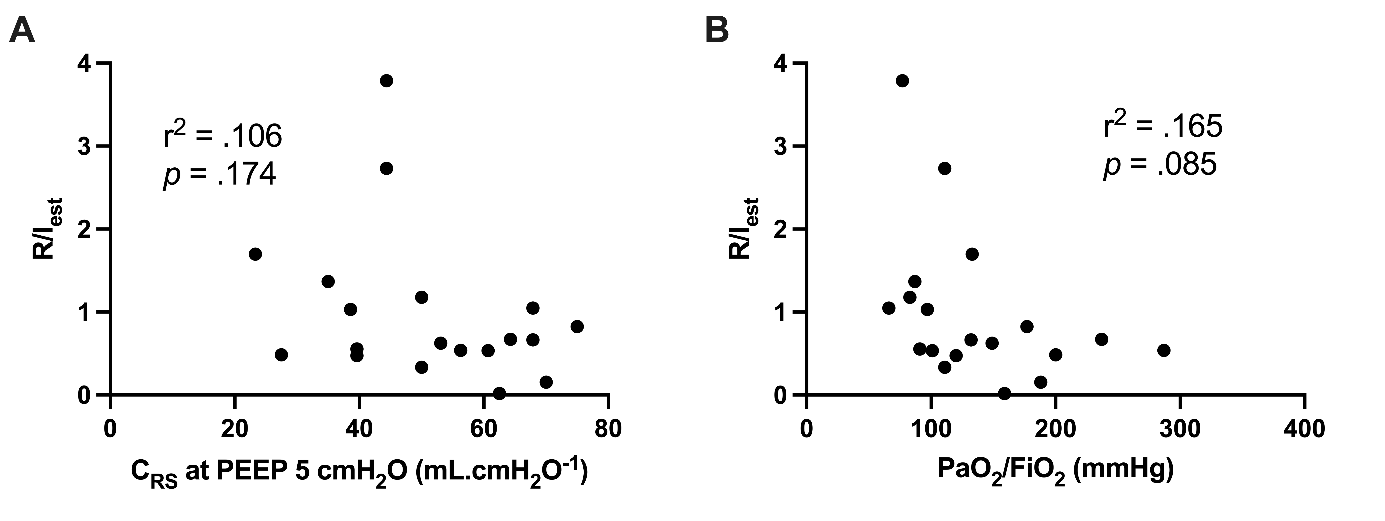
**

**Figure S9**


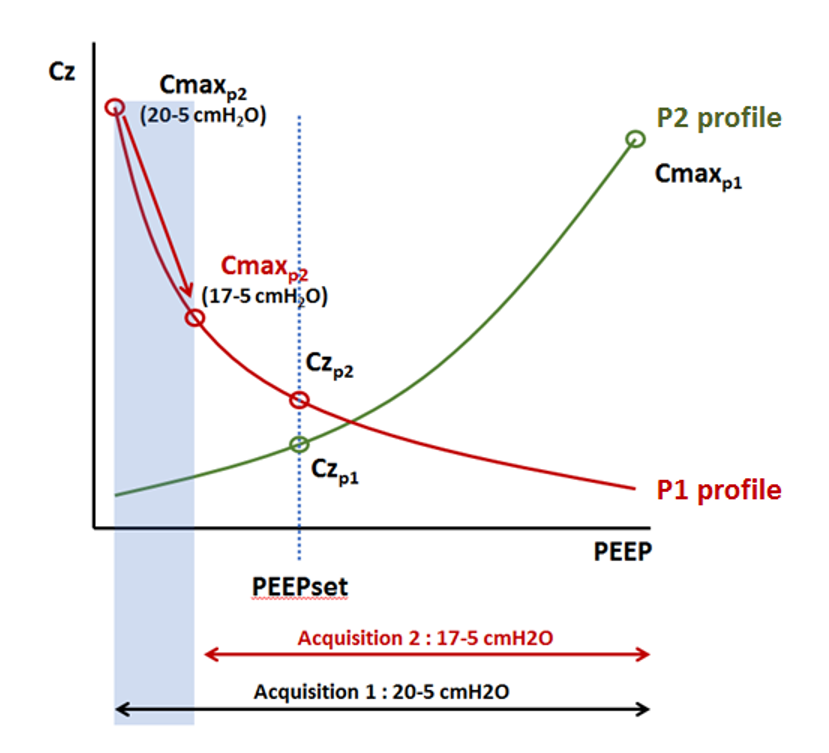

Supplement: Supplementary file 1 — Additional file 1: Table S1. Presentation and computation of physiological parameters used in the study. Figure S1. Study protocol, represented by PEEP level across time. Figure S2. Correlation between estimated recruitment-to-inflation (R/I) ratio, based on change in end-expiratory lung volume measured by electrical impedance tomography (EIT) and R/I ratio based on the single breath trial (SBT) method. Figure S3. Optimal Positive End Expiratory Pressure (PEEP) computed according to the COVID-19 status. Figure S4. Correlations between recruitability assessed by recruited volume standardized for predicted body weight (VREC/PBW) and optimal PEEP levels. Figure S5. Correlations between recruitability assessed by the difference in lung collapse between PEEP 20 and 5 cmH2O (· Collapse20-5) and optimal PEEP levels. Figure S6. Optimal Positive End-Expiratory Pressure (PEEP) level computed after the decremental PEEP trial among patients with Lower (blue boxes, n=10) and Higher (red boxes, n=9) Recruitment-to-Inflation ratio estimated by Electrical Impedance Tomography (R/Iest). Figure S7. Optimal Positive End-Expiratory Pressure (PEEP) level computed after the decremental PEEP trial. Figure S8. Correlations between respiratory system compliance (CRS, panel A) or ratio between arterial pressure in dioxygen and inspired fraction in dioxygen (PaO2/FiO2, panel B) and recruitment-to-inflation ratio estimated by Electrical Impedance Tomography (R/Iest). Figure S9. Graphic representation of the mathematical variations in lung overdistension (OD) and collapsus (LC) induced by the changes in acquisition window. [file 13613_2023_1228_MOESM1_ESM.docx]
